# Supplementary material for: Detection and Molecular Diversity of Cryptosporidium spp. and Giardia duodenalis in the Endangered Iberian Lynx (Lynx pardinus), Spain
Source: Animals (Basel). 2024 Jan 22;14(2):340. doi: 10.3390/ani14020340 (PMC10812403; doi:10.3390/ani14020340)
Supplement: Supplementary file 1 [file animals-14-00340-s001.zip › Table S1.pdf]

**Table S1**. PCR cycling conditions used for the molecular identification and/or characterization of *Cryptosporidium* spp. and *Giardia duodenalis* in the present study.

| | | Temperature and time | | | | | | |
|---|---|---|---|---|---|---|---|---|
| **Target pathogen** | **Locus** | **Initial denaturation** | **Denaturation** | **Annealing** | **Extension** | **No. cycles** | **Final extension** | **Reference** |
| *Cryptosporidium* spp. | *ssu* rRNA | 94 °C 3 min | 94 °C 40 s | 50 °C 40 s | 72 °C 1 min | 35 | 72 °C 10 min | [57] |
| *Cryptosporidium* spp. | *gp60* | 94 °C 5 min | 94 °C 45 s | 59 °C 45 s | 72 °C 1 min | 35 | 72 °C 10 min | [58] |
| *Giardia duodenalis* | *ssu* rRNA | 95 °C 15 min | 95 °C 15 s | 60 °C 1 min | 72 °C 30 s | 45 | – | [59] |
| | *ssu* rRNA | 95 °C 2 min | 95 °C 45 s | 58/55 °C 30 s | 72 °C 45 s | 35 | 72 °C 4 min | [60] |
| | *gdh* | 95 °C 3 min | 95 °C 30 s | 55 °C 30 s | 72 °C 1 min | 35 | 72 °C 7 min | [62] |
| | *bg* | 95 °C 7 min | 95 °C 30 s | 65/55 °C 30 s | 72 °C 1 min | 35 | 72 °C 7 min | [63] |
| | *tpi* | 94 °C 5 min | 94 °C 45 s | 50 °C 45 s | 72 °C 1 min | 35 | 72 °C 10 min | [64] |

*bg*: β-giardin; *gdh*: Glutamate dehydrogenase; *gp60*: 60 kDa glycoprotein; *ssu* rRNA: Small subunit ribosomal RNA; *tpi*: Triose phosphate isomerase.
