# Supplementary material for: Detection and Molecular Diversity of Cryptosporidium spp. and Giardia duodenalis in the Endangered Iberian Lynx (Lynx pardinus), Spain
Source: Animals (Basel). 2024 Jan 22;14(2):340. doi: 10.3390/ani14020340 (PMC10812403; doi:10.3390/ani14020340)
Supplement: Supplementary file 1 [file animals-14-00340-s001.zip › Table S2.pdf]

Table S2. Oligonucleotides used for the molecular identification and/or characterization of *Cryptosporidium* spp. and *Giardia duodenalis* in the present study.

Target organism	Locus	Oligonucleotide	Sequence (5´–3´)	Reference
Cryptosporidium spp.	*ssu* rRNA	CR-P1	CAGGGAGGTAGTGACAAGAA	[57]
		CR-P2	TCAGCCTTGCGACCATACTC	
		CR-P3	ATTGGAGGGCAAGTCTGGTG	
		CPB-DIAGR	TAAGGTGCTGAAGGAGTAAGG	
Cryptosporidium parvum	*gp*60	AL 3531	ATAGTCTCCGCTGTATTC	[58]
		AL 3535	GGAAGGAACGATGTATCT	
		AL 3532	TCCGCTGTATTCTCAGCC	
		AL 3534	GCAGAGGAACCAGCATC	
Giardia duodenalis	*ssu* rRNA	Probe	FAM–CCCGCGGCGGTCCCTGCTAG–BHQ1	[59]
		Gd-80F	GACGGCTCAGGACAACGGTT	
		Gd-127R	TTGCCAGCGGTGTCCG	
	ssu rRNA	Gia2029	AAGTGTGGTGCAGACGGACTC	[60]
		Gia2150c	CTGCTGCCGTCCTTGGATGT	
		RH11	CATCCGGTCGATCCTGCC	[61]
		RH4	AGTCGAACCCTGATTCTCCGCCAGG	
	gdh	GDHeF	TCAACGTYAAYCGYGGYTTCCGT	[62]
		GDHiF	CAGTACACCTCYGCTCTCGG	
		GDHiR	GTTRTCCTTGCACATCTCC	
	bg	G7_F	AAGCCCGACGACCTCACCCGCAGTGC	[63]
		G759_R	GAGGCCGCCCTGGATCTTCGAGACGAC	
		G99_F	GAACGAACGAGATCGAGGTCCG	
		G609_R	CTCGACGAGCTTCGTGTT	
	tpi	AL3543	AAATIATGCCTGCTCGTCG	[64]
		AL3546	CAAACCTTITCCGCAAACC	
		AL3544	CCCTTCATCGGIGGTAACTT	
		AL3545	GTGGCCACCACICCCGTGCC	

bg: β-giardin; *gdh*: Glutamate dehydrogenase; *ssu* rRNA: Small subunit ribosomal RNA; *tpi*: Triose phosphate isomerase.
